# Quantification of active bearing input force for vibration reduction performance of unbalanced rotor systems

**DOI:** 10.1038/s41598-023-35993-w

**Published:** 2023-06-02

**Authors:** Dongwoo Hong, Hyeongill Lee, Youkyung Han, Byeongil Kim

**Affiliations:** 1 School of Mechanical Engineering, Yeungnam University, Gyeongsan, 38541 Republic of Korea; 2 School of Automotive Engineering, Kyungpook National University, Sangju, 37224 South Korea; 3 Department of Civil Engineering, Seoul National University of Science and Technology, Seoul, 01811 South Korea

**Keywords:** Engineering, Mechanical engineering

## Abstract

Recently, rotating machinery has been widely applied in various mechanical systems such as hydroelectric and nuclear power plants. When mechanical systems are operated, the main rotor is rotated to manufacture the product. If a fault occurs in the rotor, then the system is damaged. Thus, to avoid malfunction of the system and rotor damage, vibration issues because of bending, misalignment, and imbalance should be considered. In this regard, a smart structure-based active bearing system is extensively researched and developed to control rotor vibration. This system can continuously improve the noise, vibration, and harshness performance under various operating conditions by controlling the dynamic characteristics of the active bearing. This study focused on the effect of rotor motion control by quantifying the active bearing force and phase when an active bearing was applied in a simple rotor model. A simple rotor with two active bearing systems was modeled based on lumped-parameter modeling. In the rotor model, the active bearing, which had two piezoelectric actuators and rubber grommets placed in both the x- and y-directions, was located on both sides to control the vibration. The interaction between the rotor and the active bearing system was considered to quantify the force and phase of this system. Furthermore, through simulation, the motion control effect was validated when an active bearing was applied in the rotor model.

## Introduction

Recently, rotating machinery has been widely applied in various mechanical systems to manufacture products and generate electrical power. When mechanical systems are operated, the main rotor part is rotated, exciting the overall system. If a fault occurs in the main rotor part, such as bending, misalignment, or imbalance, the overall system malfunctions or is damaged. In order to avoid these issues, vibration control should be performed for the main rotor. For achieving this, a smart structure-based active bearing system has been widely researched and developed. This system can continuously improve noise, vibration, and harshness performance under various operating conditions by controlling the dynamic characteristics of the active bearing system.

Several contemporary studies have focused on rotating machinery. The finite element method (FEM)^1–3^ and the transfer matrix^4,5^ method are widely used to analyze rotor systems, and the lumped-parameter modeling is less frequently used. Werner^6,7^ modeled asynchronous machines based on lumped-parameter modeling, compared the results with those of FEM; they observed that lumped-parameter modeling had higher accuracy. However, in the lumped-parameter model, the response point is fixed, whereas it can be selected by adjusting the number of elements in FEM or the transfer matrix method. Thus, this limitation must be overcome. Werner^8,9^ performed vibration control for an induction motor based on an actuator located between the motor feet and soft foundation, and they showed that the vibration was efficiently reduced.

Active bearing systems with smart materials, such as magnetic and piezoelectric actuators, have been actively investigated to control rotor vibration^10^. In several studies, active magnetic bearings (AMBs) have been used. Lusty et al. proposed an internal-stator active magnetic actuator (AMA) for vibration reduction of a hollow rotating shaft and demonstrated a vibration reduction effect through the experiment^11^. Chen et al. proposed an AMB and bearingless motor to stabilize a conventional rotor system^12^. Saeed et al. performed rotor AMB system control by combining proportional–derivative (PD) and positive position feedback controllers and showed that the system lateral vibration was close to zero^13^. Bordoloi et al. used a genetic algorithm to optimize the control of the AMB system and achieved vibration reduction^14^. Yao et al. proposed an AMA to control a rotor system based on PD control^15^. The simulation and experiment demonstrated the effectiveness of the proposed method for vibration reduction.

Piezoelectric actuators have been used in several studies. Zhang et al. proposed a mixed sensitivity robust controller for flexible rotors with piezoelectric actuators^16^. Jungblut et al. performed active vibration control through an experimental modal analysis based on a piezoelectric actuator and achieved a vibration reduction effect^17^. Heinedl et al. proposed a control algorithm based on the Lyapunov stability theorem to eliminate the imbalance and resonance^18^. Li et al. proposed a novel active control method based on a piezoelectric actuator that successfully suppressed milling chatter^19^. To reduce the cost of the active vibration control system and simplify the structure, piezoelectric self-sensing actuators has been widely applied in control systems^20,21^. In addition, a piezoelectric patch was applied to the rotor surface to reduce the vibration of the rotor bearing system^22^.

In the above-mentioned literatures on the modeling, FEM and TMM shows a great coincidence with experimental data for analyzing the rotor response. However, in case of those methods, $n\times n$ matrices must be processed, so it takes fairly large amount of time to obtain responses due to the high calculation burden. In addition, when the model is changed, the entire matrix configuration must be newly defined, which is cumbersome. On the other hand, if we use the lumped parameter method, the calculation speed is faster compared to both FEM and TMM, and it has the advantage of being able to receive a response immediately when the model parameters are changed. Werner^8,9^ analyzed the overall responses by modeling each part of rotor machinery system through lumped parameter method, while this approach cannot determine responses at a certain point along the shaft which is possible with FEM and TMM. However, research to overcome this limitation and see the response at an arbitrary location has not yet been performed. Also, from the above-mentioned literature on active bearing systems, contemporary studies have mainly focused on active control algorithms. Therefore, this paper focused on two parts, as follows: (1) check the response at an arbitrary point on the shaft modeled by the lumped parameter method, and (2) quantify the force and phase of an active bearing system. In order to see the detailed response of the shaft in the rotating system modeled by the lumped parameter method, a transfer matrix based on the internally dividing point method was proposed and the response was confirmed. In addition, to quantify the force and phase of the active bearing system, the relationship between the active bearing and the rotor system was considered and calculated. If the input signal of the actuator can be quantified, the amount of voltage used during control can be predicted, and furthermore, it can be used as an index for optimal positioning of the active bearing system based on the mode shape and quantified force. In this paper, it is organized by focusing only on quantification, and the control voltage prediction and optimal location selection will be dealt with later. The rotating speed was set to 400 rpm to confirm the feasibility of the vibration reduction performance in the driving condition of typical hydroelectric power plants. Furthermore, for the validation of the proposed method in various conditions, control performance was investigated at three different operating speeds. A rotor system with two active bearings was modeled based on a lumped-parameter model. The active bearing, which had piezoelectric actuators and rubber grommets placed in both the x- and y-directions, was located on both sides to control the vibration. When using FEM or experiments, the sensor position can be selected more arbitrarily, whereas it is rather impossible when using the lumped-parameter model. To overcome this limitation of lumped-parameter modeling, a coordinate transformation method is suggested. The active bearing force and phase are quantified considering the relationship between the rotor and active bearing system and assuming that the bearing housing motion is zero. The main contributions of this study can be summarized as follows: (1) lumped-parameter modeling was performed for rotor systems with active bearings; (2) to overcome the limitation in the lumped-parameter model, which chooses the response point, the coordinate transform matrix was established; and (3) the active bearing force and phase were quantified to control the rotor system.

The remainder of this paper is organized as follows. “Mathematical modeling” section describes lumped-parameter modeling for a rotor with two active bearing systems and explains the transformation matrix. “Quantification of active bearing force and phase” section describes the quantification method of the force and phase for the active bearing system. “Validation of rotor motion control” section discusses the motion control results, and finally, “Conclusion” section presents the conclusion and discusses future work.

## Mathematical modeling

**Figure 1:**
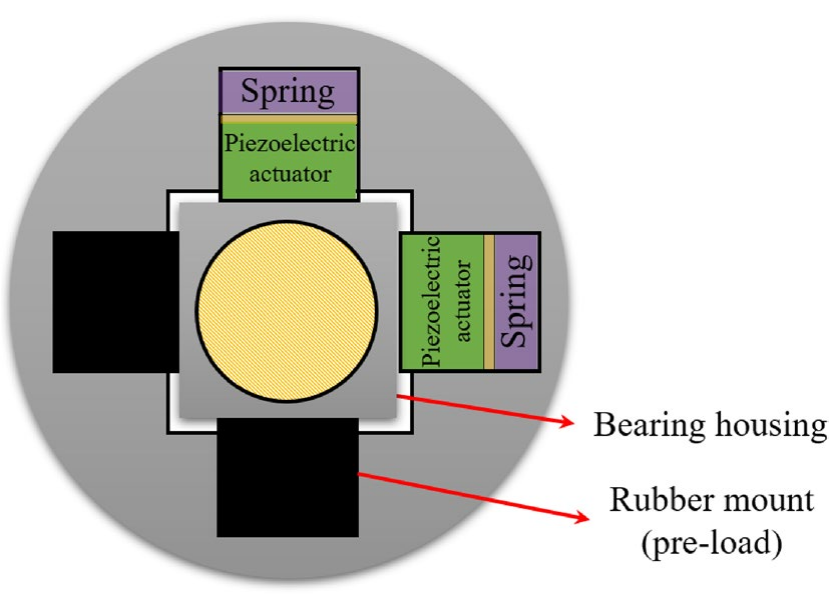
Schematic of active bearing system.

A simple rotor system with two active bearing parts was modeled based on the lumped-parameter model to validate the vibration reduction effect by quantifying the active bearing force and phase. A schematic of the active bearing system, consisting of a piezoelectric actuator and rubber grommet in the x- and y-directions, is shown in Fig. 1.

**Figure 2:**
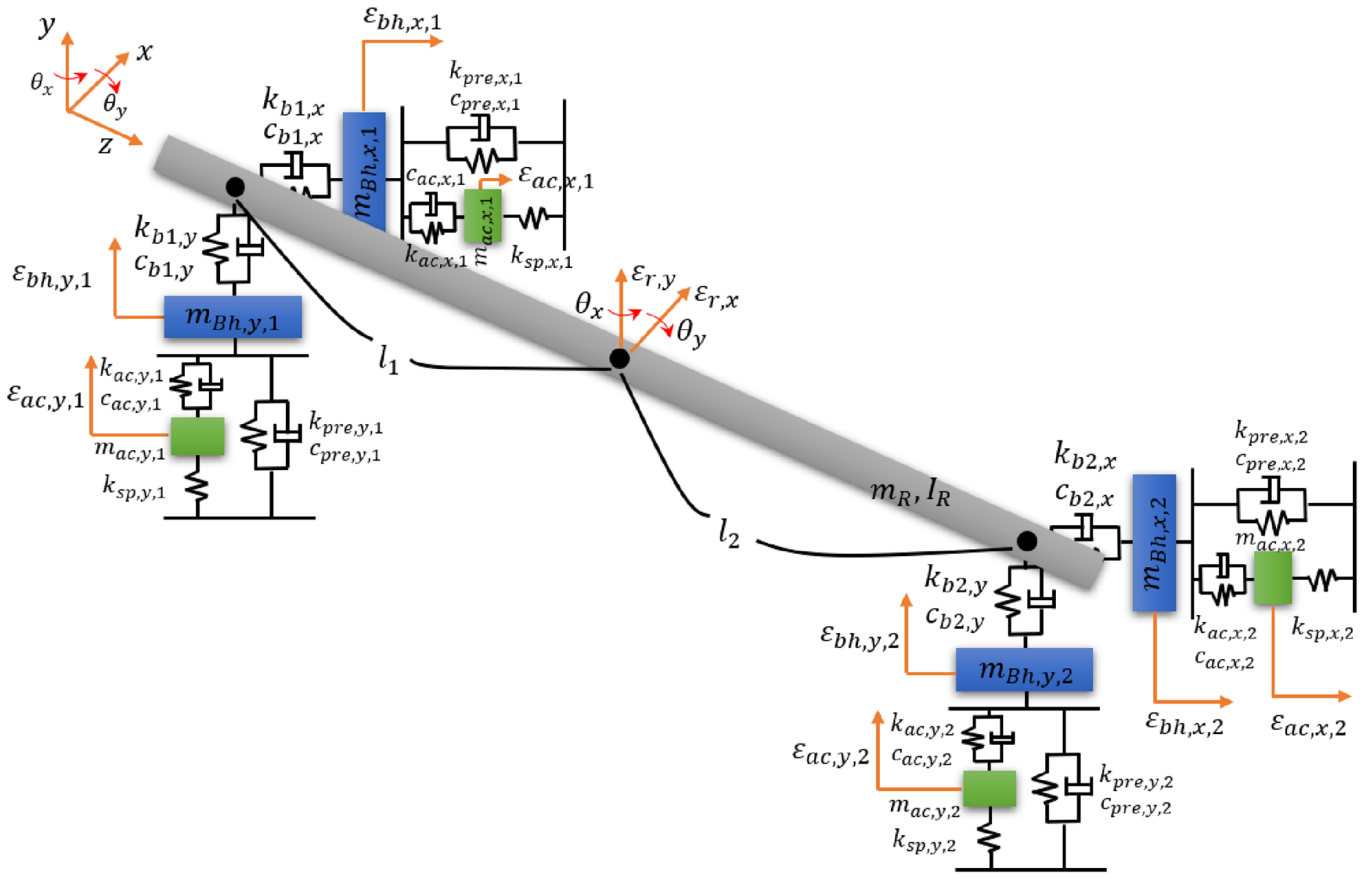
Simple rotor model with two active bearing systems.

Figure 2 shows the overall model with a shaft (made of SM45C) whose diameter and length are 0.01 m and 0.2 m, respectively. Material properties of all the components are obtained from a real laboratory setup. The active bearing system having a piezoelectric actuator and rubber mount is located on both sides to control the vibration. ${m}_{R}$ and ${I}_{R}$ represent the mass and inertia of the shaft, respectively. ${m}_{Bh,n,i}$ and ${m}_{as,n,i}$ are the masses of the bearing housing and piezoelectric actuator, respectively, where $n=x,y$, and $i=\mathrm{1,2}$. The rotor mass and inertia were calculated based on the material property, and the mass of the bearing housing and piezoelectric actuator was measured and used. ${k}_{bi,n}$ and ${c}_{bi,n}$ represent the stiffness and damping coefficients corresponding to the bearing, respectively. When a ball or roller bearing is considered, the damping coefficient is assumed to be zero. In this study, the ball bearing was used when a simulation was performed; thus, the damping coefficient was not considered. ${k}_{ac,n,i}$ and ${c}_{ac,n,i}$ represent the stiffness and damping coefficients of the piezoelectric actuator, respectively. ${k}_{sp,n,i}$ is the stiffnesses of the spring. ${k}_{pre,n,i}$ and ${c}_{pre,n,i}$ represent the stiffness and damping coefficients of the rubber grommet, respectively.

The stiffness and damping coefficient for the actuator and rubber grommet were assumed to follow the Kelvin-Voigt model. ${\varepsilon }_{r,n}$ and ${\theta }_{n}$ represent the translational and rotational motions of the rotor for each n-direction, where n = x and y. ${\varepsilon }_{bh,n,i}$ and ${\varepsilon }_{ac,n,i}$ are the translational motions of the bearing housing and piezoelectric actuator, respectively. The overall parameters are listed in Table 1. Matrices M, C, and K for x- and y-directions are summarized in (1)–(5). The displacements ${q}_{n}$ and unbalanced forces ${F}_{n}$ are summarized in (6)–(9).

**Table 1:** Rotor parameters.

Variable		Values	Unit
Operating speed		400	RPM
Rotor (SM45C)	Young’s modulus (E)	207	GPa
	Density ($\rho$)	7600	kg/m^3^
	length ($${l}_{i})$$	0.2	m
Bearing	Stiffness (${k}_{bi,n} i=\mathrm{1,2}, n=x,y)$	$2\times {10}^{8}$	N/m
	Housing (${m}_{bh,n,i} i=\mathrm{1,2}, n=x,y)$	0.133	kg
Actuator	Mass (${m}_{ac,n,i} i=\mathrm{1,2}, n=x,y)$	0.196	kg
	Stiffness (${k}_{ac,n,i} i=\mathrm{1,2}, n=x,y$)	5.64(1 + i0.034)	KN/mm
Spring stiffness (${k}_{sp,n,i} i=\mathrm{1,2}, n=x,y)$		$27.24\times {10}^{6}$	N/m
Rubber grommet (${k}_{pre,n,i} i=\mathrm{1,2}, n=x,y$)		0.61(1 + i0.300)	KN/mm

1 $${M}_{y}={M}_{x}=diag\left(\left[\begin{array}{cccccc}{m}_{R}& {m}_{B1}& {m}_{ac1}& {m}_{B2}& {m}_{ac2}& {I}_{R}\end{array}\right]\right)$$

2 $${K}_{y}=\left[\begin{array}{cccccc}{k}_{b1,y}+{k}_{b2,y}& -{k}_{b1,y}& 0& -{k}_{b2,y}& 0& {k}_{b1,y}{l}_{1}-{k}_{b2,y}{l}_{2}\\ -{k}_{b1,y}& {k}_{b1,y}+{k}_{ac,y,1}+{k}_{pre}& -{k}_{ac,y,1}& 0& 0& -{k}_{b1,y}{l}_{1}\\ 0& -{k}_{ac,y,1}& {k}_{ac,y,1}+{k}_{sp}& 0& 0& 0\\ -{k}_{b2,y}& 0& 0& {k}_{b2,y}+{k}_{ac,y,2}+{k}_{pre}& -{k}_{ac,y,2}& {k}_{b2,y}{l}_{2}\\ 0& 0& 0& -{k}_{ac,y,2}& {k}_{ac,y,1}+{k}_{sp}& 0\\ {k}_{b1,y}{l}_{1}-{k}_{b2,y}{l}_{2}& -{k}_{b1,y}{l}_{1}& 0& {k}_{b2,y}{l}_{2}& 0& {k}_{b1,y}{l}_{1}^{2}+{k}_{b2,y}{l}_{2}^{2}+{k}_{r}\end{array}\right]$$

3 $${C}_{y}=\left[\begin{array}{cccccc}{c}_{b1,y}+{c}_{b2,y}& -{c}_{b1,y}& 0& -{c}_{b2,y}& 0& {c}_{b1,y}{l}_{1}-{c}_{b2,y}{l}_{2}\\ -{c}_{b1,y}& {c}_{b1,y}+{c}_{ac,y,1}+{c}_{pre}& -{c}_{ac,y,1}& 0& 0& -{c}_{b1,y}{l}_{1}\\ 0& -{c}_{ac,y,1}& {c}_{ac,y,1}+{c}_{sp}& 0& 0& 0\\ -{c}_{b2,y}& 0& 0& {c}_{b2,y}+{c}_{ac,y,2}+{c}_{pre}& -{c}_{ac,y,2}& {c}_{b2,y}{l}_{2}\\ 0& 0& 0& -{c}_{ac,y,2}& {c}_{ac,y,1}+{c}_{sp}& 0\\ {c}_{b1,y}{l}_{1}-{c}_{b2,y}{l}_{2}& -{c}_{b1,y}{l}_{1}& 0& {c}_{b2,y}{l}_{2}& 0& {c}_{b1,y}{l}_{1}^{2}+{c}_{b2,y}{l}_{2}^{2}\end{array}\right]$$

4 $${K}_{x}=\left[\begin{array}{cccccc}{k}_{b1,x}+{k}_{b2,x}& -{k}_{b1,x}& 0& -{k}_{b2,x}& 0& -{k}_{b1,x}{l}_{1}+{k}_{b2,x}{l}_{2}\\ -{k}_{b1,x}& {k}_{b1,x}+{k}_{ac,x,1}+{k}_{pre}& -{k}_{ac,x,1}& 0& 0& {k}_{b1,x}{l}_{1}\\ 0& -{k}_{ac,x,1}& {k}_{ac,x,1}+{k}_{sp}& 0& 0& 0\\ -{k}_{b2,x}& 0& 0& {k}_{b2,x}+{k}_{ac,x,2}+{k}_{pre}& -{k}_{ac,x,2}& -{k}_{b2,x}{l}_{2}\\ 0& 0& 0& -{k}_{ac,x,2}& {k}_{ac,x,1}+{k}_{sp}& 0\\ -{k}_{b1,x}{l}_{1}+{k}_{b2,x}{l}_{2}& {k}_{b1,x}{l}_{1}& 0& -{k}_{b2,x}{l}_{2}& 0& {k}_{b1,x}{l}_{1}^{2}+{k}_{b2,x}{l}_{2}^{2}+{k}_{r}\end{array}\right]$$

5 $${C}_{x}=\left[\begin{array}{cccccc}{c}_{b1,x}+{c}_{b2,x}& -{c}_{b1,x}& 0& -{c}_{b2,x}& 0& -{c}_{b1,x}{l}_{1}+{c}_{b2,x}{l}_{2}\\ -{c}_{b1,x}& {c}_{b1,x}+{c}_{ac,x,1}+{c}_{pre}& -{c}_{ac,x,1}& 0& 0& {c}_{b1,x}{l}_{1}\\ 0& -{c}_{ac,x,1}& {c}_{ac,x,1}+{c}_{sp}& 0& 0& 0\\ -{c}_{b2,x}& 0& 0& {c}_{b2,x}+{c}_{ac,x,2}+{c}_{pre}& -{c}_{ac,x,2}& -{c}_{b2,x}{l}_{2}\\ 0& 0& 0& -{c}_{ac,x,2}& {c}_{ac,x,1}+{c}_{sp}& 0\\ -{c}_{b1,x}{l}_{1}+{c}_{b2,x}{l}_{2}& {c}_{b1,x}{l}_{1}& 0& -{c}_{b2,x}{l}_{2}& 0& {c}_{b1,x}{l}_{1}^{2}+{c}_{b2,x}{l}_{2}^{2}\end{array}\right]$$

6 $${q}_{y}={\left[\begin{array}{cccccc}{\varepsilon }_{r,y}& {\varepsilon }_{bh,y,1}& {\varepsilon }_{ac,y,1}& {\varepsilon }_{bh,y,2}& {\varepsilon }_{ac,y,2}& {\theta }_{x}\end{array}\right]}^{T}$$

7 $${q}_{x}={\left[\begin{array}{cccccc}{\varepsilon }_{r,x}& {\varepsilon }_{bh,x,1}& {\varepsilon }_{ac,x,1}& {\varepsilon }_{bh,x,2}& {\varepsilon }_{ac,x,2}& {\theta }_{y}\end{array}\right]}^{T}$$

8 $${F}_{y}={\left[\begin{array}{cccccc}{f}_{un,y}& 0& 0& 0& {f}_{ac,y}& 0\end{array}\right]}^{T}$$

9 $${F}_{x}={\left[\begin{array}{cccccc}{f}_{un,x}& 0& 0& 0& {f}_{ac,x}& 0\end{array}\right]}^{T}$$

In (9), ${f}_{un,n}$ and ${f}_{ac,n}$ represent the unbalanced and actuator forces, respectively. The overall equation of motion for the rotor system can be expressed by (10).

10 $$\left[\begin{array}{cc}{M}_{y}& 0\\ 0& {M}_{x}\end{array}\right]\left[\begin{array}{c}{\ddot{q}}_{y}\\ {\ddot{q}}_{x}\end{array}\right]+\left[\begin{array}{cc}{C}_{y}& \Omega I\\ -\Omega I& {C}_{x}\end{array}\right]\left[\begin{array}{c}{\dot{q}}_{y}\\ {\dot{q}}_{x}\end{array}\right]+\left[\begin{array}{cc}{K}_{y}& 0\\ 0& {K}_{x}\end{array}\right]\left[\begin{array}{c}{q}_{y}\\ {q}_{x}\end{array}\right]=\left[\begin{array}{c}{F}_{y}\\ {F}_{x}\end{array}\right]$$

**Figure 3:**
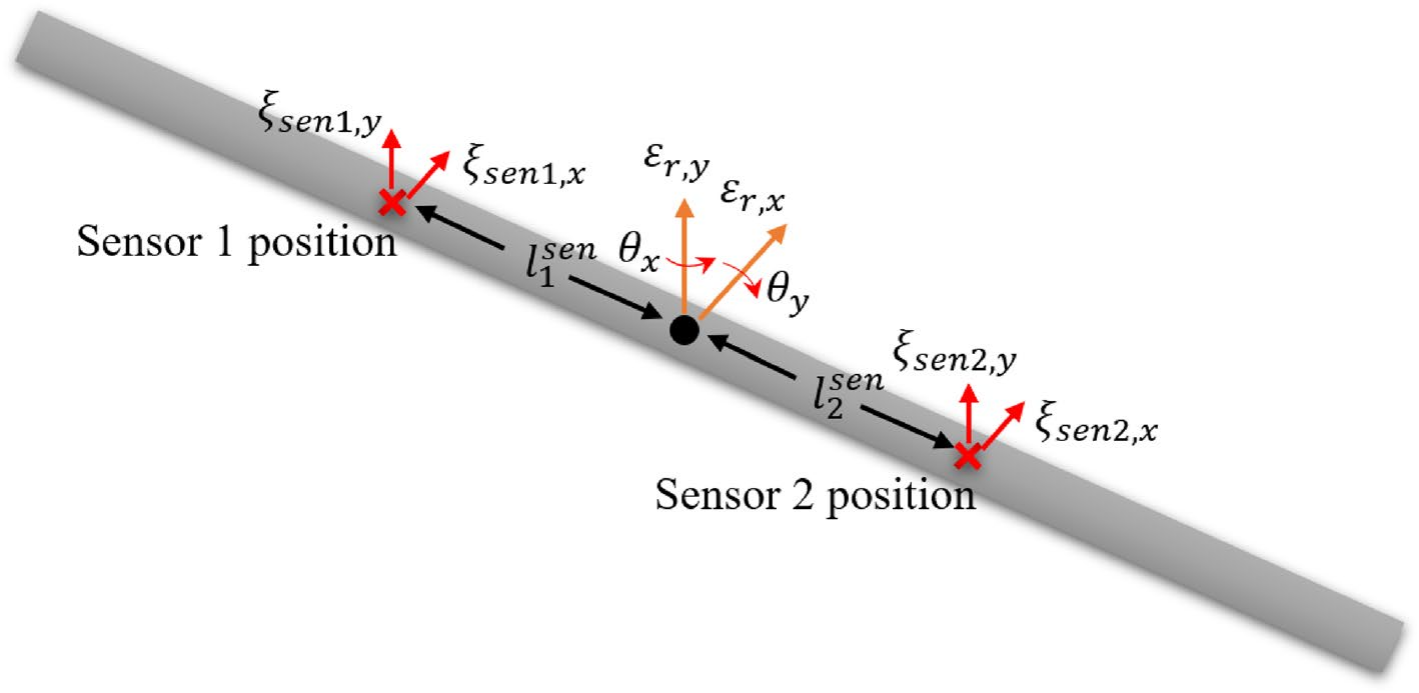
Sensor position in shaft part.

When the rotor system is analyzed using FEM or experiments, the sensor position can be arbitrarily selected. However, (10) considers the center of mass of the rotor; therefore, the response point (sensor position) is fixed with no choice in the lumped-parameter model. To overcome this limitation, a coordinate transform is performed using the transformation matrix Π, and the shaft part in Fig. 1 is re-expressed, as shown in Fig. 3. FEM or TMM require re-calculation of matrices to simulate the condition of parametric change and different response positions of the shaft, which takes considerably long time due to the large amount of calculation. In the case of the lumped parameter method, responses can be immediately checked through a simple parameter change without changing the model with less amount of calculation. In addition, through the coordinate conversion method proposed in this paper, the response at any point along the shaft can be easily obtained by changing the variable ${l}_{i}^{sen}$ determining the response position.

In Fig. 3, ${\xi }_{seni,n}$ represents the displacement corresponding to the ith sensor in the n-direction, and ${l}_{i}^{sen}$ is the length corresponding to the center of mass and the sensor location, where i = 1, 2, and n = x and y. To divide the center of mass into each sensor location, the transformation matrix $\Pi$ is defined in (11), and the time-invariant and the rotational motion are assumed to be small, ${\theta }_{n}\le 1$.

11 $$\Pi =\left[\begin{array}{cccccccccccc}\frac{{l}_{2}^{sen}}{{l}_{1}^{sen}+{l}_{2}^{sen}}& \frac{{l}_{1}^{sen}}{{l}_{1}^{sen}+{l}_{2}^{sen}}& 0& 0& 0& 0& 0& 0& 0& 0& 0& 0\\ 0& 0& 1& 0& 0& 0& 0& 0& 0& 0& 0& 0\\ 0& 0& 0& 1& 0& 0& 0& 0& 0& 0& 0& 0\\ 0& 0& 0& 0& 1& 0& 0& 0& 0& 0& 0& 0\\ 0& 0& 0& 0& 0& 1& 0& 0& 0& 0& 0& 0\\ \frac{-1}{{l}_{1}^{sen}+{l}_{2}^{sen}}& \frac{1}{{l}_{1}^{sen}+{l}_{2}^{sen}}& 0& 0& 0& 0& 0& 0& 0& 0& 0& 0\\ 0& 0& 0& 0& 0& 0& \frac{{l}_{2}^{sen}}{{l}_{1}^{sen}+{l}_{2}^{sen}}& \frac{{l}_{1}^{sen}}{{l}_{1}^{sen}+{l}_{2}^{sen}}& 0& 0& 0& 0\\ 0& 0& 0& 0& 0& 0& 0& 0& 1& 0& 0& 0\\ 0& 0& 0& 0& 0& 0& 0& 0& 0& 1& 0& 0\\ 0& 0& 0& 0& 0& 0& 0& 0& 0& 0& 1& 0\\ 0& 0& 0& 0& 0& 0& 0& 0& 0& 0& 0& 1\\ 0& 0& 0& 0& 0& 0& \frac{-1}{{l}_{1}^{sen}+{l}_{2}^{sen}}& \frac{1}{{l}_{1}^{sen}+{l}_{2}^{sen}}& 0& 0& 0& 0\end{array}\right]$$

Using (11), the displacement can be rewritten as (12) and (13) through the relationship of $q=\Pi q\mathrm{^{\prime}}$.

12 $${q}_{y}^{\mathrm{^{\prime}}}={\left[\begin{array}{cccccc}{\xi }_{sen1,y}& {\xi }_{sen2,y}& {\varepsilon }_{bh,y,1}& {\varepsilon }_{ac,y,1}& {\varepsilon }_{bh,y,2}& {\varepsilon }_{ac,y,2}\end{array}\right]}^{T}$$

13 $${q}_{x}^{\mathrm{^{\prime}}}={\left[\begin{array}{cccccc}{\xi }_{sen1,x}& {\xi }_{sen2,x}& {\varepsilon }_{bh,x,1}& {\varepsilon }_{ac,x,1}& {\varepsilon }_{bh,x,2}& {\varepsilon }_{ac,x,2}\end{array}\right]}^{T}$$

Based on this assumption, the sensor position can be selected by changing the ${l}_{i}^{sen}$, and the overall equation of motion can be rewritten as (14), which represents the sensor position.

14 $$\left[\begin{array}{cc}{M}_{y}^{\mathrm{^{\prime}}}& 0\\ 0& {M}_{x}^{\mathrm{^{\prime}}}\end{array}\right]\left[\begin{array}{c}{\ddot{q}}_{y}^{\mathrm{^{\prime}}}\\ {\ddot{q}}_{x}^{\mathrm{^{\prime}}}\end{array}\right]+\left[\begin{array}{cc}{C}_{y}^{\mathrm{^{\prime}}}& \Omega I\\ -\Omega I& {C}_{x}^{\mathrm{^{\prime}}}\end{array}\right]\left[\begin{array}{c}{\dot{q}}_{y}^{\mathrm{^{\prime}}}\\ {\dot{q}}_{x}^{\mathrm{^{\prime}}}\end{array}\right]+\left[\begin{array}{cc}{K}_{y}^{\mathrm{^{\prime}}}& 0\\ 0& {K}_{x}^{\mathrm{^{\prime}}}\end{array}\right]\left[\begin{array}{c}{q}_{y}^{\mathrm{^{\prime}}}\\ {q}_{x}^{\mathrm{^{\prime}}}\end{array}\right]=\left[\begin{array}{c}{F}_{y}\\ {F}_{x}\end{array}\right]$$

Furthermore, (14) can be rewritten as (15).

15 $${M}^{\mathrm{^{\prime}}}\ddot{Q}\mathrm{^{\prime}}+{C}^{\mathrm{^{\prime}}}\dot{Q}\mathrm{^{\prime}}+K\mathrm{^{\prime}}Q\mathrm{^{\prime}}=F$$

## Quantification of active bearing force and phase

**Figure 4:**
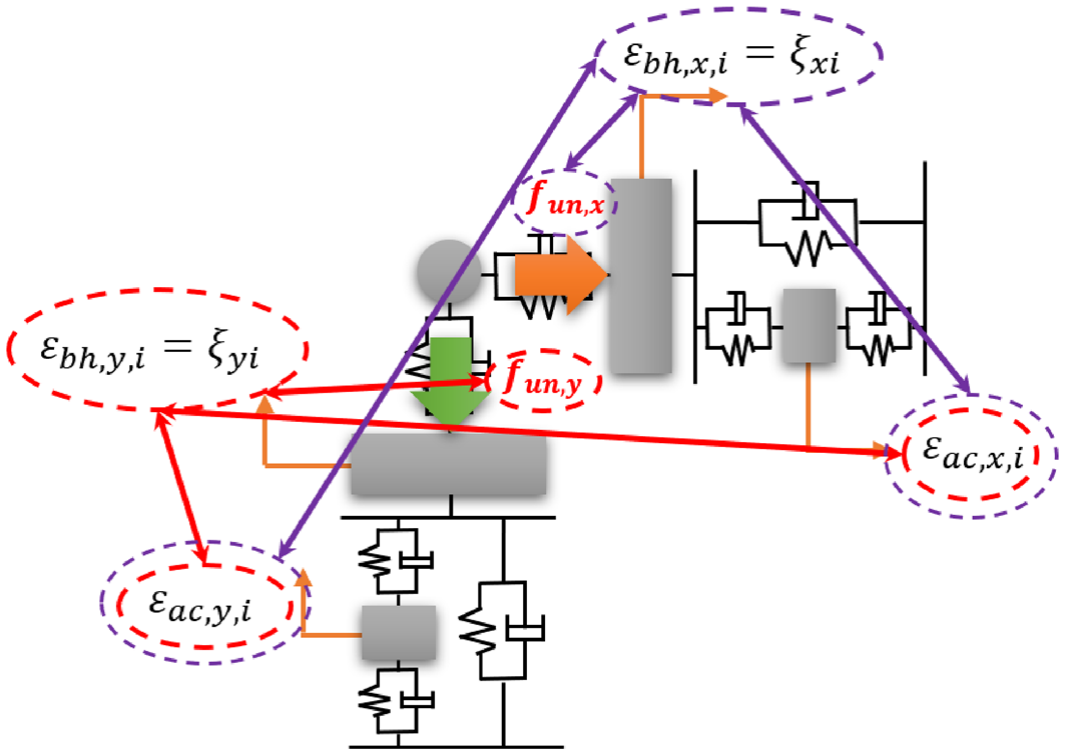
Relationship between external force and system motion.

To validate the vibration reduction performance through an active bearing system, the input signals were quantified considering the relationship between the external force and system motion, as shown in Fig. 4.

In Fig. 4, red and purple line represent the relationship between external force and system motion corresponding to y- and x-direction, respectively. Each bearing housing in x- and y-direction is excited by external force ${f}_{un,y}$ and ${f}_{un,x}$, and it is creating the bearing housing motion ${\varepsilon }_{bh,y,i}$ and ${\varepsilon }_{bh,x,i}$. In order to control the housing motion that occurs by external force, the piezoelectric actuator generates ${\varepsilon }_{ac,y,i}$ and ${\varepsilon }_{ac,x,i}$. For ideal control, it is assumed that the housing motion is zero for each direction. When the shaft is operated, the bearing housing vibrations have x- and y-components, which are not independent. Thus, when considering the x- and y-direction motions for the bearing housing, the effects of the two actuators should be also considered. Thus, when considering the housing motion in the y direction, not only the external force and actuator motion in the y direction, but also the actuator motion in the x direction should be considered. This fact also holds in the x-direction. When the relationship is considered, the phase between the harmonic excitation and system motion is critical. Thus, to consider the phase, complex valued variables were assumed for excitation and actuator force, as in (16) to (19).

16 $${f}_{un,y}(t)={m}_{o}e{\Omega }^{2}{e}^{i\Omega t}$$

17 $${f}_{un,x}(t)={m}_{o}e{\Omega }^{2}{e}^{i\Omega t}$$

18 $${f}_{aci,y}(t)={F}_{aci,y}{e}^{i\left(\Omega t+{\phi }_{aci,y}\right)}$$

19 $${f}_{aci,x}(t)={F}_{aci,x}{e}^{i\left(\Omega t+{\phi }_{aci,x}\right)}$$

In (16)–(19), ${m}_{o}$ and e represent the unbalanced mass and eccentricity, respectively, $\Omega$ is the rotation speed, and ${F}_{aci,n}$ and ${\phi }_{aci,n}$ are the force and phase corresponding to the ith actuator in the n-direction, respectively, where i = 1, 2, and n = x and y. To simplify the notation of bearing housing, the notations were changed to ${\varepsilon }_{bh,y,i}={\xi }_{yi}$ and ${\varepsilon }_{bh,x,i}={\xi }_{xi}$. For effective vibration isolation of the rotor, the bearing housing motion should ideally be minimized to zero. Thus, the bearing housing motion was defined by (20) and (21):

20 $${\xi }_{yi}=\left({\Xi }_{bh,y,i,wy}+{\Xi }_{bh,y,i,ac,x,ij}{e}^{i{\phi }_{ac,x,ij}}+{\Xi }_{bh,y,i,ac,y,ij}{e}^{i{\phi }_{ac,y,ij}}\right){e}^{i\Omega t}$$

21 $${\xi }_{xi}=\left({\Xi }_{bh,x,i,wx}+{\Xi }_{bh,x,i,ac,x,ij}{e}^{i{\phi }_{ac,x,ij}}+{\Xi }_{bh,x,i,ac,y,ij}{e}^{i{\phi }_{ac,y,ij}}\right){e}^{i\Omega t}$$

In (20) and (21), ${\Xi }_{bh,y,i,wy}$ and ${\Xi }_{bh,x,i,wx}$ are the complex amplitudes in the y- and x-directions corresponding to the ith bearing housing due to the unbalance forces in y- and x-directions, respectively, where i = 1, 2. ${\Xi }_{bh,y,i,ac,n,ij}$ and ${\Xi }_{bh,x,i,ac,n,ij}$ are the complex amplitudes in the y- and x-directions, respectively, corresponding to the ith bearing housing due to jth actuator, where i and j = 1 and 2 and n = x and y. ${\phi }_{ac,x,ij}$ and ${\phi }_{ac,y,ij}$ are the phases in x- and y-directions, respectively, corresponding to ith bearing housing and jth actuator. The bearing housing motion has two actuator terms in the other direction. When the rotor is operated, the bearing housing vibrations have x- and y-components, which are not independent. Thus, when considering the x- and y-direction motions for the bearing housing, the effects of the two actuators should be considered. Ideally, phase match should be conducted for one state, such as external force or for each actuator. Furthermore, the amplitude $\Xi$ should be zero. Thus, to fit the phase match, (20) and (21) were rewritten in terms of magnitude and phase, as in (22) and (23):

22 $${\xi }_{yi}=\left(\left|{\Xi }_{bh,y,i,wy}\right|{e}^{i{\beta }_{bh,y,i,wy}}+\left|{\Xi }_{bh,y,i,ac,x,ij}\right|{e}^{i\left({\beta }_{bh,y,i,ac,x,ij}+{\phi }_{ac,x,ij}\right)}+\left|{\Xi }_{bh,y,i,ac,y,ij}\right|{e}^{i\left({\beta }_{bh,y,i,ac,y,ij}+{\phi }_{ac,y,ij}\right)}\right){e}^{i\Omega t}$$

23 $${\xi }_{xi}=\left(\left|{\Xi }_{bh,x,i,wx}\right|{e}^{i{\beta }_{bh,x,i,wx}}+\left|{\Xi }_{bh,x,i,ac,x,ij}\right|{e}^{i\left({\beta }_{bh,x,i,ac,x,ij}+{\phi }_{ac,x,ij}\right)}+\left|{\Xi }_{bh,x,i,ac,y,ij}\right|{e}^{i\left({\beta }_{bh,x,i,ac,y,ij}+{\phi }_{ac,y,ij}\right)}\right){e}^{i\Omega t}$$

In (22) and (23), $\left|\cdot \right|$ represents the magnitude operator. ${\beta }_{bh,y,i,wy}$ is the phase between the unbalanced force and motion of the ith bearing housing, both in the y-direction, where i = 1, 2. ${\beta }_{bh,x,i,wx}$ is the phase between the unbalanced force and motion of the ith bearing housing, both in the x-direction, where i = 1, 2. ${\beta }_{bh,y,i,ac,n,ij}$ is the phase between the jth actuator force in the n-direction and the motion of the ith bearing housing in the y-direction, where i and j = 1, 2 and n = x and y. ${\beta }_{bh,x,i,ac,n,ij}$ is the phase between the jth actuator force in the n-direction and motion of the ith bearing housing in the x-direction, where i and j = 1, 2, and n = x and y. To perform motion control, phase matching should be conducted, but (22) and (23) have five phases. Thus, a phase match should be performed to determine the relationship between the unbalanced force and bearing housing motion. Through this assumption, an out-of-phase motion can be created. Using the relationship between the bearing housing and active bearing system, (24) and (25) were defined, which represent the x- and y-directions, respectively:

24 $${\beta }_{bh,y,i,wy}={\beta }_{bh,y,i,ac,x,ij}+{\phi }_{ac,x,ij}, \; {\beta }_{bh,y,i,wy}={\beta }_{bh,y,i,ac,y,ij}+{\phi }_{ac,y,ij} \; (\text{y-direction})$$

25 $${\beta }_{bh,x,i,wx}={\beta }_{bh,x,i,ac,x,ij}+{\phi }_{ac,x,ij}, \; {\beta }_{bh,x,i,wx}={\beta }_{bh,x,i,ac,y,ij}+{\phi }_{ac,y,ij} \;(\text{x-direction})$$

To summarize the phase term, (24) and (25) were redefined as ${\phi }_{ac,x,ij}$ and ${\phi }_{ac,y,ij}$ in (26) and (27):

26 $${\phi }_{ac,x,ij}={\beta }_{bh,y,i,wy}-{\beta }_{bh,y,i,ac,x,ij}, \; {\phi }_{ac,y,ij}={\beta }_{bh,y,i,wy}-{\beta }_{bh,y,i,ac,y,ij} \; (\text{y-direction})$$

27 $${\phi }_{ac,x,ij}={\beta }_{bh,x,i,wx}-{\beta }_{bh,x,i,ac,x,ij}, \;  {\phi }_{ac,y,ij}={\beta }_{bh,x,i,wx}-{\beta }_{bh,x,i,ac,y,ij} \; (\text{x-direction})$$

Equations (22) and (23) can be rewritten as (28) and (29) by substituting ${\phi }_{ac,x,ij}$ and ${\phi }_{ac,y,ij}$, showing the phase match corresponding to the unbalanced force and bearing housing.

28 $${\xi }_{yi}=\left(\left|{\Xi }_{bh,y,i,wy}\right|+\left|{\Xi }_{bh,y,i,ac,x,ij}\right|+\left|{\Xi }_{bh,y,i,ac,y,ij}\right|\right){e}^{i\left(\Omega t+{\beta }_{bh,y,i,wy}\right)}$$

29 $${\xi }_{xi}=\left(\left|{\Xi }_{bh,x,i,wx}\right|+\left|{\Xi }_{bh,x,i,ac,x,ij}\right|+\left|{\Xi }_{bh,x,i,ac,y,ij}\right|\right){e}^{i\left(\Omega t+{\beta }_{bh,x,i,wx}\right)}$$

This study focused on reducing rotor vibration through an active bearing system by applying quantified force and phase. To conduct motion control, the active bearing forces were quantified through the defined bearing housing motion. Thus, each magnitude value was calculated using the compliance matrix $H(\Omega )$. The dynamic stiffness matrix $\kappa \mathrm{^{\prime}}$ was used to calculate $H(\Omega )$ and is defined as ${\kappa }^{\mathrm{^{\prime}}}\left(\Omega \right)=-{M}^{\mathrm{^{\prime}}}{\omega }^{2}+{C}^{\mathrm{^{\prime}}}j\omega +K$. The compliance matrix $H(\Omega )$ is defined by (30):

30 $$H\left(\Omega \right)=\left[\begin{array}{cccc}{H}_{1 1}& {H}_{1 2}& \cdots & {H}_{1 12}\\ {H}_{2 1}& {H}_{2 2}& \cdots & {H}_{2 12}\\ \vdots & \vdots & \ddots & \vdots \\ {H}_{12 1}& {H}_{12 2}& \dots & {H}_{12 12}\end{array}\right]$$

Using the compliance matrix, system motion $Q$, and external force, the system displacement is defined by (31).

31 $$Q\left(\Omega \right){e}^{i\Omega t}=H\left(\Omega \right)F\left(\Omega \right){e}^{i\Omega t}$$

where $Q$ is the system motion and $F$ represents the external force, including the unbalanced and active bearing forces. The magnitude values in (20) and (21) were calculated using (31), and each amplitude was calculated using (32)–(35).

32 $${\Xi }_{bh,y,1,wy}={H}_{3\,1}{f}_{un,y}, \; {\Xi }_{bh,y,1,ac,x,11}={H}_{3\,3}{f}_{ac,y}, \; {\Xi }_{bh,y,1,ac,y,11}={H}_{3\,9}{f}_{ac,x}$$

33 $${\Xi }_{bh,y,2,wy}={H}_{5\,1}{f}_{un,y}, \;  {\Xi }_{bh,y,2,ac,x,22}={H}_{5\,5}{f}_{ac,y }, \;{\Xi }_{bh,y,2,ac,y,22}={H}_{5\,11}{f}_{ac,x}$$

34 $${\Xi }_{bh,x,1,wy}={H}_{9\,7}{f}_{un,x}, \; {\Xi }_{bh,x,1,ac,x,11}={H}_{9\,3}{f}_{ac,y}, \; {\Xi }_{bh,x,1,ac,y,11}={H}_{9\,9}{f}_{ac,x}$$

35 $${\Xi }_{bh,x,2,wy}={H}_{11\,7}{f}_{un,x}, \; {\Xi }_{bh,x,2,ac,x,22}={H}_{11\,5}{f}_{ac,y}, \; {\Xi }_{bh,x,2,ac,y,22}={H}_{11\,11}{f}_{ac,x}$$

Furthermore, to calculate the phase for each amplitude, the phase operator $\mathrm{\angle }$ was applied and expressed as ${\beta }_{bh,y,1,wy}=\mathrm{\angle }{H}_{3 1}$. The other phases were calculated using the same method. Using the amplitude and phase values, the active bearing force was quantified using (28) and (29), assuming the magnitude term to be zero, as defined in (36) and (37).

36 $$\left|{\Xi }_{bh,y,i,wy}\right|+\left|{\Xi }_{bh,y,i,ac,x,ij}\right|+\left|{\Xi }_{bh,y,i,ac,y,ij}\right|=0$$

37 $$\left|{\Xi }_{bh,x,i,wx}\right|+\left|{\Xi }_{bh,x,i,ac,x,ij}\right|+\left|{\Xi }_{bh,x,i,ac,y,ij}\right|=0$$

Through (36) and (37), the active bearing system forces were defined as (38) and (39):

38 $$\left[\begin{array}{c}{F}_{ac1,y}\\ {F}_{ac1,x}\end{array}\right]=-\frac{1}{{H}_{33}{H}_{99}-{H}_{39}{H}_{93}}\left[\begin{array}{cc}-{H}_{9\,9}& {H}_{3\,9}\\ {H}_{9\,3}& {-H}_{3\,3}\end{array}\right]\left[\begin{array}{c}{H}_{3\,1}{f}_{un,y}\\ {H}_{9\,7}{f}_{un,x}\end{array}\right]$$

39 $$\left[\begin{array}{c}{F}_{ac2,y}\\ {F}_{ac2,x}\end{array}\right]=-\frac{1}{{H}_{55}{H}_{11\,11}-{H}_{5\,11}{H}_{11\,5}}\left[\begin{array}{cc}-{H}_{11\,11}& {H}_{5\,11}\\ {H}_{11\,5}& {-H}_{5\,5}\end{array}\right]\left[\begin{array}{c}{H}_{5\,1}{f}_{un,y}\\ {H}_{11\,7}{f}_{un,x}\end{array}\right]$$

## Validation of rotor motion control

**Figure 5:**
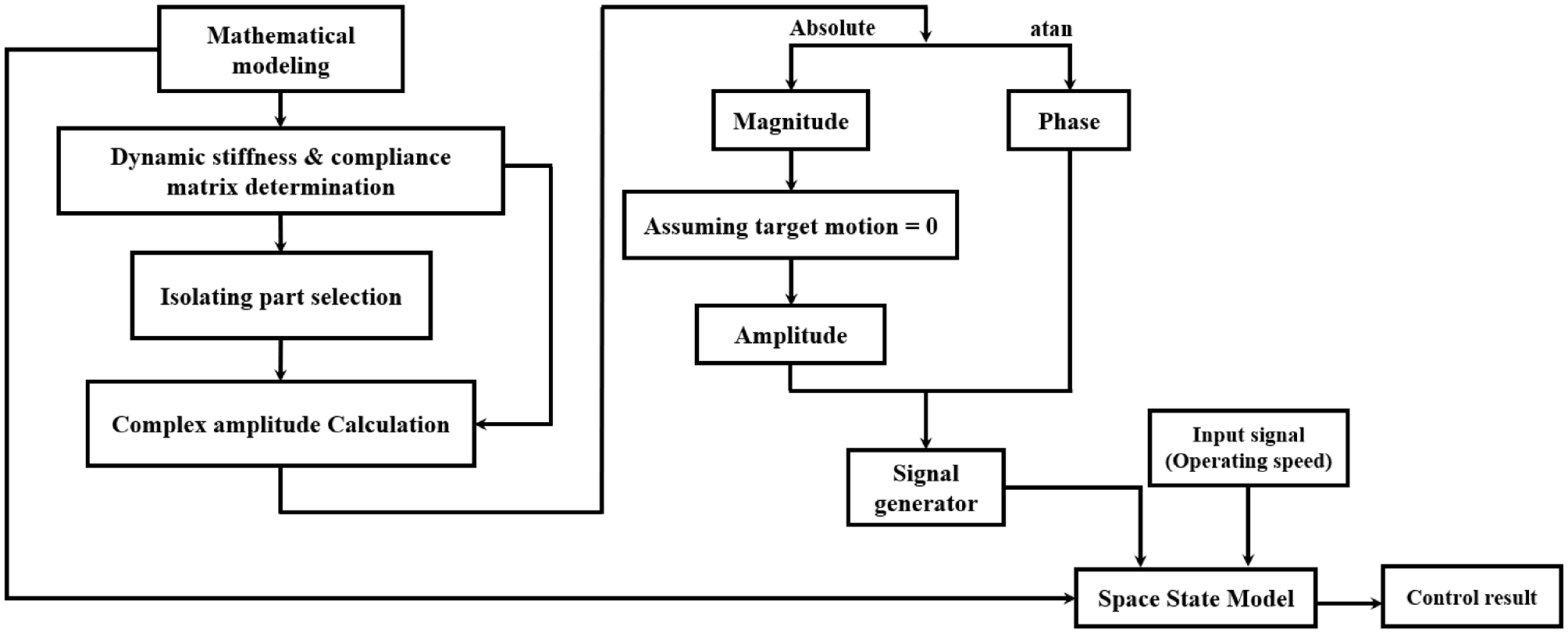
Flowchart about proposed method.

The flowchart for checking the vibration reduction performance through the proposed quantification method is shown in Fig. 5 below. First, mathematical modeling is done on the entire model, and dynamic stiffness and compliance matrix are calculated. And then, the vibration isolation target is selected and the complex amplitudes of the isolated part are calculated. Magnitude and phase component is extracted and the control force, the input signal for active bearing system, is calculated based on the magnitude after assuming that the motion of the target part becomes zero. Finally, a signal is generated based on the derived control force magnitude and phase, and then used as an input to the active bearing system.

To validate the effect of vibration reduction performance, a simulation was performed. The external force was set to an unbalanced force in the x- and y-directions, and the sampling frequency was 10 kHz. In addition, the equation of motion was expressed using the state-space model and is summarized in (40) and (41).

40 $${\dot{x}}^{\mathrm{^{\prime}}}(t)={\varvec{A}}x\mathrm{^{\prime}}(t)+{\varvec{B}}u(t)$$

41 $$y(t)={\varvec{C}}x\mathrm{^{\prime}}(t)+{\varvec{D}}u(t)$$

In (40) and (41), A, B, and C are the system state, input, and output matrices, respectively, as summarized in (42).

42 $$A=\left[\begin{array}{cc}{O}_{n\times n}& {I}_{n\times n}\\ -K\mathrm{^{\prime}}/M\mathrm{^{\prime}} & -C\mathrm{^{\prime}}/M\mathrm{^{\prime}}\end{array}\right],\;B=\left[\begin{array}{c}{O}_{n\times n}\\ 1/M\mathrm{^{\prime}}\end{array}\right], \; C=\left[\begin{array}{cc}{I}_{n\times n}& {O}_{n\times n}\end{array}\right], \; D=\left[{O}_{n\times n}\right]$$

A simulation was performed using the state-space model. In addition, the shaft part was divided by 5 points on both the left and right sides based on the center of mass, and the vibration reduction performance was checked.

**Figure 6:**
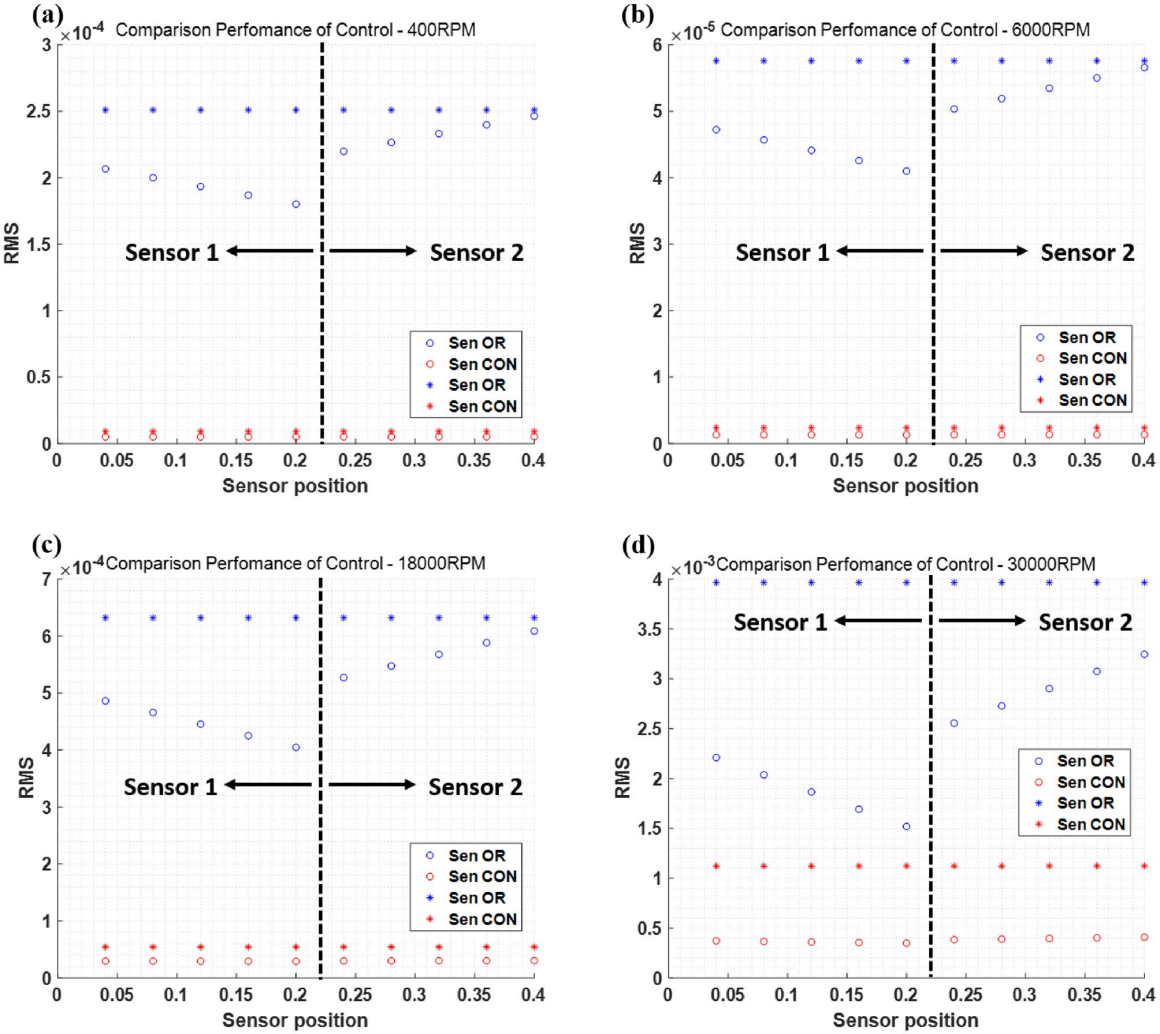
Comparison of control performance—RMS value; (**a**) 400RPM, (**b**) 6000RPM, (**c**) 18000RPM, (**d**) 30000RPM.

Root mean square (RMS), insertion loss (IL), and whirling motions were used to validate the simulation results. When performing the simulation, in order to validate the control performance at different operating speed, four cases are tested. Fig. 6 shows the RMS value for each point, and Table 2 lists the average RMS values for each sensor part. Since each sensor part has two displacement sensors on x- and y-directions, the RMS values are calculated for both of them and compared before and after control for the validation of vibration reduction performance. In Fig. 6, the black dotted line represents the rotor center and the blue marks stand for the original (before control) values, while the red marks indicate the controlled (after control) values. In addition, * and ο marks represent RMS values corresponding to x- and y-direction, respectively. The time signal was used when the RMS value was calculated.

**Table 2:** Control performance comparison—RMS value.

Comparison control performance ($RMS*{10}^{-4}$)		X-direction		Y-direction	
		Sensor 1	Sensor 2	Sensor 1	Sensor 2
400RPM	Original	$2.508\times {10}^{-4}$	$2.508\times {10}^{-4}$	$1.935\times {10}^{-4}$	$2.332\times {10}^{-4}$
	Control	$0.0903\times {10}^{-4}$	$0.0903\times {10}^{-4}$	$0.050\times {10}^{-4}$	$0.051\times {10}^{-4}$
Reduction rate (%)		$96.40\%\downarrow$	$96.40\%\downarrow$	$97.42\%\downarrow$	$97.82\%\downarrow$
6000RPM	Original	$5.757\times {10}^{-5}$	$5.757\times {10}^{-5}$	$4.411\times {10}^{-5}$	$5.344\times {10}^{-5}$
	Control	$2.3571\times {10}^{-6}$	$2.3596\times {10}^{-6}$	$1.312\times {10}^{-6}$	$1.335\times {10}^{-6}$
Reduction rate (%)		$95.91\%\downarrow$	$95.91\%\downarrow$	$97.02\%\downarrow$	$97.50\%\downarrow$
18000RPM	Original	$6.322\times {10}^{-4}$	$6.322\times {10}^{-4}$	$4.455\times {10}^{-4}$	$5.680\times {10}^{-4}$
	Control	$5.446\times {10}^{-5}$	$5.443\times {10}^{-5}$	$2.940\times {10}^{-5}$	$3.018\times {10}^{-5}$
Reduction rate (%)		$91.39\%\downarrow$	$91.39\%\downarrow$	$93.49\%\downarrow$	$94.68\%\downarrow$
30000RPM	Original	0.004	0.004	0.0019	0.0029
	Control	0.0011	0.0011	$3.612\times {10}^{-4}$	$3.970\times {10}^{-4}$
Reduction rate (%)		$72.5\%\downarrow$	$72.5\%\downarrow$	$80.99\%\downarrow$	$86.31\%\downarrow$

Figure 6 and Table 2 show the rotor control performance when the quantified force and phase were used as the active bearing inputs. In the x- and y-directions, the vibration was significantly reduced. However, as the rotational speed increases, the vibration reduction effect caused by the active bearing tends to decrease. Thus, to validate the reduction performance, the IL was used, defined in (43).

43 $$IL \left[dB\right]=20\mathrm{log}\left(\frac{before \; control}{after \; control}\right)$$

In (43), the values before and after control are determined based on the compliance matrix and quantified force and phase, as defined in (32) to (35). Using this relationship, IL was calculated, and the results are summarized in Table 3.

**Table 3:** Control performance comparison—RMS value.

Comparison control performance (insertion loss [$\mathrm{dB}]$)		X-direction		Y-direction	
		Sensor 1	Sensor 2	Sensor 1	Sensor 2
Insertion loss	400RPM	72.24	93.42	72.26	88.29
	6000RPM	48.25	73.92	77.57	63.32
	18000RPM	36.61	71.8	70.76	54.12
	30000RPM	24.54	48.29	48	51.01

**Figure 7:**
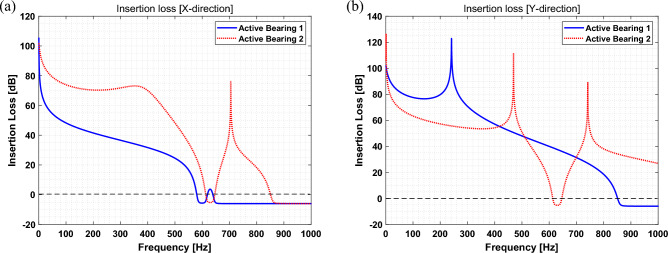
Insertion loss for (**a**) x- and (**b**) y-direction.

**Figure 8:**
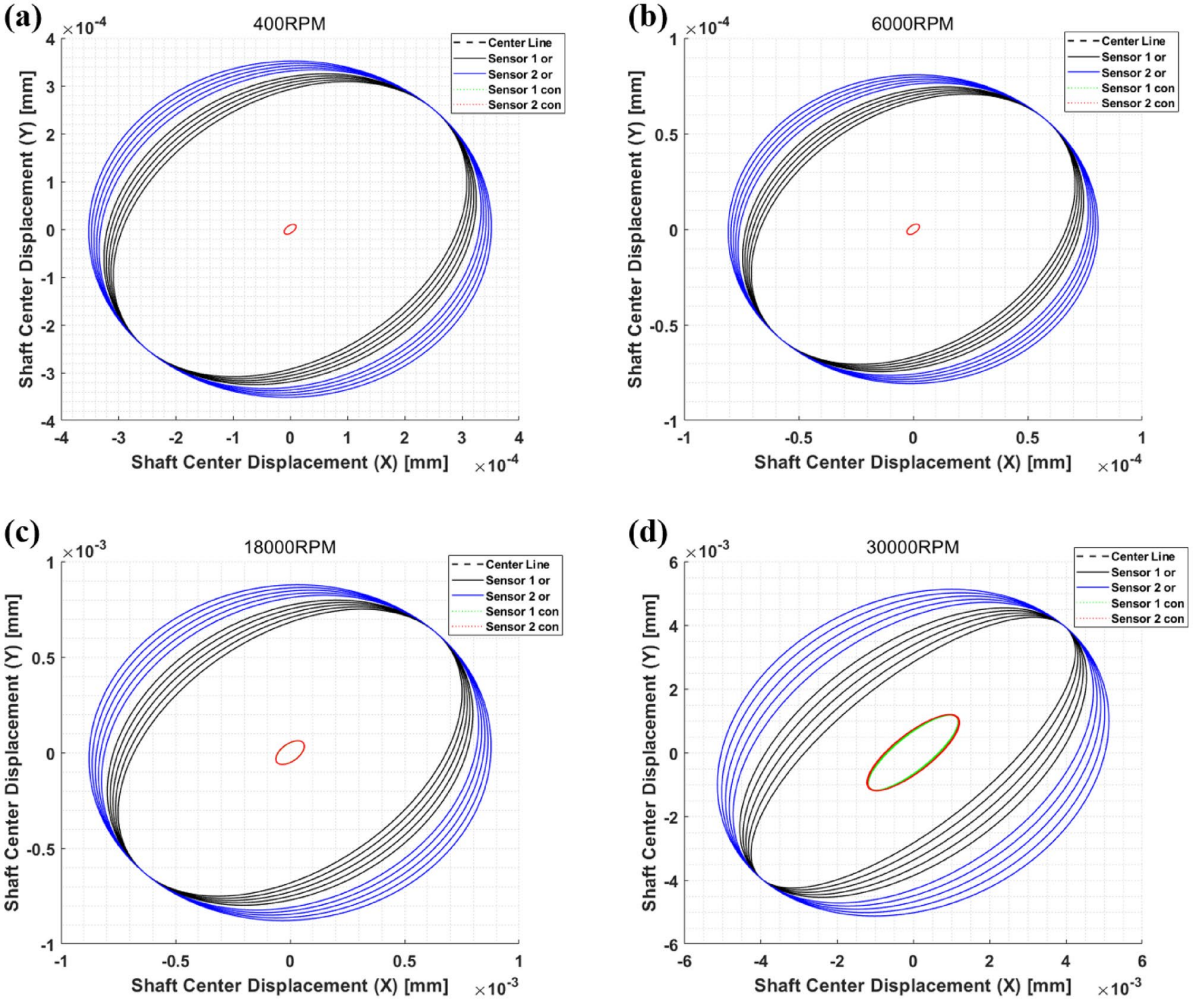
Comparison of control performance—(**a**) 400RPM, (**b**) 6000RPM, (**c**) 18000RPM, (**d**) 30000RPM.

The IL had a positive value, indicating a decrease in each sensor position. Based on the calculated IL in Table 3, it can be seen that the vibration is effectively reduced in both the x- and y-directions when the active bearing system is installed at both ends of the shaft. In addition, as the rotation speed increases, the trend of insertion loss decreases, and through this, it can be verified that the vibration reduction performance by the active bearing decreases as the rotation speed increases. Furthermore, the controllability of the system can be confirmed using the IL by changing RPM (Hz), as shown in Fig. 7. In Fig. 7, the black dotted line represents the control criteria. A high IL indicates a good performance, whereas a low value indicates a poor performance. Thus, the peak value exhibited the desired performance. In addition, an IL value lower than the block-dotted line indicates that the controlled signal has a higher value than the uncontrolled signal. Based on IL results, it can be seen that both the x- and y-directions show good performance from 0 to 550 Hz (33,000 RPM), and then the control performance drops. As the operating speed increases, vibration reduction performance by active bearings tends to be decreased and the reason can be summarized as follows. Based on Fig. 6 and Table 2, it can be seen that the displacement of the shaft increases as the rotational speed increases. Since the piezoelectric actuator has limited specification on the displacement, it can be expected that the vibration reduction performance would be worse at the operating speed with relatively high displacement. Thus, when motion control is performed by applying quantified force and phase, the controllable level can be determined considering the shaft displacement and actuator specification. Furthermore, a parametric study of the variables that can be changed in the rotor system, such as the stiffness, damping coefficient, and bearing location, is possible. A follow-up study will be conducted in the future. Finally, to confirm the rotor whirling motion through the active bearing, the whirling motion at each point is shown in Fig. 8, where the black and blue lines represent the whirling motion corresponding to the uncontrolled state, and the green and red dotted lines represent the whiling motion corresponding to the controlled state through the active bearing system.

When rotor motion control was conducted based on the quantified force and phase, the rotor whirling motion for each point was significantly reduced. Thus, the active bearing with a piezoelectric actuator had a good performance based on the above results. Furthermore, the limitation of lumped-parameter modeling was overcome through the transformation matrix. Using this method, the result for each point can be analyzed, enabling the calculation of time and simplifying the rotor model. Based on these results, the performance of the rotor motion control is proven when applying the quantified signal and the transformation matrix in a rotor system with an active bearing model.

## Conclusion

In this study, to validate the rotor motion control through the active bearing system, the force and phase were quantified considering the relationship between the rotor and active bearing system. The main contributions of this study are as follows: (1) a rotor system consisting a shaft with two active bearing systems was modeled based on lumped-parameter modeling, (2) a transformation matrix was suggested to overcome the limitation of the lumped-parameter model, and (3) the input force and phase of the active bearing system were quantified considering the relationship between the rotor and active bearing system.

A study was conducted to reduce the overall rotor motion for a simple rotor model. Thus, to control the rotor motion, a simple shaft with two active bearing systems was modeled based on lumped-parameter modeling. Although in this model the response point (sensor position) is fixed, other methods, such as the FEM or the transfer matrix method, can be used to select the sensor position. Thus, to overcome this limitation, a transformation matrix was suggested. Using this matrix, the response point (sensor position) can be selected in the shaft. In addition, to perform motion control, the force and phase were quantified by considering the relationship between the rotor and active bearing system. The equation of motion was expressed in the state-space model to perform the simulation. When the simulation was performed, the quantified force and phase were applied to the active bearing system. Through simulation, the performance of the rotor motion control was demonstrated. The proposed method can be applied to determine the optimized variable and bearing position by changing parameters. Furthermore, it can be applied to other structures with mounting systems. In the future, to perform real-time control, an adaptive algorithm, such as the least mean square algorithm and `neural-network-based signal tracking algorithm, will be applied in active bearing systems. In addition, the optimized location for the active bearing was determined based on the relationship between the mode shape and quantified force to perform optimized control. Finally, this approach is being applied to more complicated systems, such as wind turbine and vehicle engine.

## Data Availability

The datasets used and/or analysed during the current study available from the corresponding author on reasonable request.
